# Identification of FAT3 as a new candidate gene for adolescent idiopathic scoliosis

**DOI:** 10.1038/s41598-022-16620-6

**Published:** 2022-07-19

**Authors:** Dina Nada, Cédric Julien, Simon Papillon-Cavanagh, Jacek Majewski, Mohamed Elbakry, Wesam Elremaly, Mark E. Samuels, Alain Moreau

**Affiliations:** 1 Viscogliosi Laboratory in Molecular Genetics of Musculoskeletal Diseases, Sainte-Justine University Hospital Research Center, (room 2.17.027), 3175 Chemin de la Côte-Ste-Catherine, Montreal, QC H3T 1C5 Canada; 2 Pharmacology and Biochemistry Department, Faculty of Pharmacy, The British University in Egypt, Cairo, Egypt; 3 Injury Repair Recovery Program, McGill University Health Center Research Institute, Montreal, QC Canada; 4 Department of Human Genetics, McGill University, Montreal, QC Canada; 5 Biochemistry Division, Chemistry Department, Faculty of Science, Tanta University, Tanta, Egypt; 6 Department of Biochemistry and Molecular Medicine, Faculty of Medicine, Université de Montréal, Montreal, QC Canada; 7 Sainte-Justine University Hospital Research Center, Montreal, QC Canada; 8 Department of Medicine, Faculty of Medicine, Université de Montréal, Montreal, QC Canada; 9 Department of Stomatology, Faculty of Dentistry, Université de Montréal, Montreal, QC Canada

**Keywords:** Genetics, Diseases

## Abstract

In an effort to identify rare alleles associated with adolescent idiopathic scoliosis (AIS) whole-exome sequencing was performed on a discovery cohort of 73 unrelated patients and 70 age-and sex matched controls, all of French-Canadian ancestry. A collapsing gene burden test was performed to analyze rare protein-altering variants using case–control statistics. Since no single gene achieved statistical significance, targeted exon sequencing was performed for 24 genes with the smallest *p* values, in an independent replication cohort of unrelated severely affected females with AIS and sex-matched controls (N = 96 each). An excess of rare, potentially protein-altering variants was noted in one particular gene, *FAT3*, although it did not achieve statistical significance. Independently, we sequenced the exomes of all members of a rare multiplex family of three affected sisters and unaffected parents. All three sisters were compound heterozygous for two rare protein-altering variants in *FAT3*. The parents were single heterozygotes for each variant. The two variants in the family were also present in our discovery cohort. A second validation step was done, using another independent replication cohort of 258 unrelated AIS patients having reach their skeletal maturity and 143 healthy controls to genotype nine *FAT3* gene variants, including the two variants previously identified in the multiplex family: p.L517S (rs139595720) and p.L4544F (rs187159256). Interestingly, two *FAT3* variants, rs139595720 (genotype A/G) and rs80293525 (genotype C/T), were enriched in severe scoliosis cases (4.5% and 2.7% respectively) compared to milder cases (1.4% and 0.7%) and healthy controls (1.6% and 0.8%). Our results implicate *FAT3* as a new candidate gene in the etiology of AIS.

## Introduction

Adolescent Idiopathic Scoliosis (AIS) is a complex disorder of the spine, and the most common form of such disorders. It is a three-dimensional deformity of the spine characterized by a lateral curvature of ≥ 10° on a standing radiograph (Cobb method), combined with vertebral rotation. It mostly occurs at the age of adolescence and affects 1–4%^1^ of the global pediatric population with higher prevalence in females who are generally more severely affected than males^2^. In most cases the underlying cause of idiopathic scoliosis is unknown, although a genetic component is well recognized^3,4^. Twin and family studies have documented high rates of concordance among twins and increased risk to relatives of patients with AIS^5,6^. The mode of inheritance is still unclear^7^. The genetic nature of the disease is complex, with an apparent high level of heterogeneity between different families^8–10^. A number of candidate genes and loci have been suggested by different studies, but few have been successfully replicated^11^. Human genetic studies have used both linkage and association methods. The results of linkage studies have been poorly reproducible^11^. Genome wide association studies (GWAS) have identified several candidate genes for AIS including *CHL1*, *LBX1*, *GPR126*, *BNC2*, and *PAX1*^12–16^. The associated common single nucleotide polymorphisms (SNPs) identified to date only explain a small portion of the genetic component of the disease. Genetic interactions^17^ and rare variants^18^ might explain part of this “missing heritability” in AIS^19^.

Few studies have attempted to detect rare causal variants in AIS and this field of research is still in its infancy. Sequencing using either whole exome or targeted gene panels, has identified several genes that might contribute to the occurrence and or severity of scoliosis; such as *FBN1*, *FBN2*^20^, *HSPG2*^21^, *POC5*^22^, and *AKAP2*^23^. Another study suggested that accumulation of rare variants in a group of genes of the extracellular matrix might contribute to disease risk^24^. In summary, genome-wide association studies (GWAS) cannot reveal all genetic determinants associated with AIS, which is true with other complex traits. Such limitation is not exclusive to GWAS, as no method or technology to date can identify all the genetic components of complex traits despite the fact that candidate gene approach tends to have greater statistical power than studies that use large numbers of single nucleotide. Overall, this explains why the genetic component of AIS is not yet fully understood, leaving significant room for further research.

In this study, we performed whole-exome sequencing (WES) in a French-Canadian AIS cohort, followed by a targeted sequencing of the 24 statistically-strongest candidate genes from WES, in an independent replication cohort. In parallel, we performed WES in a unique multiplex family of three affected sisters with healthy parents. Our goal was to identify new genes enriched with rare variants, which might contribute to the disease. Our results implicate a novel gene, *FAT3*, not previously associated with AIS, as a strong candidate for this condition.

## Results

### Study populations

Our discovery cohort includes 73 unrelated AIS patients (68 females and 5 males), and 70 sex- and age-matched controls, all of French-Canadian ancestry (Table 1). Fifty of the patients were considered severely affected as their Cobb angles were at least 40°, and the remaining patients were considered as moderate cases (10°–39°). Our first independent replication cohort includes 96 unrelated AIS patients (only females) and 96 healthy controls (only females), which we used for the replication of the top 24 genes from the discovery cohort (Table 2). Our second replication cohort includes 258 unrelated AIS patients (82.9% females), who have reached their skeletal maturity and stratified by spinal deformity severity (Cobb angle ≥ 40° versus Cobb angle < 40°), and 143 healthy controls (Table 3).

**Table 1:** Clinical and demographic characteristics of participants in the discovery cohort.

AIS patients		Healthy controls
Age (years)	Highest Cobb Angle (°)	Age (years)
**All**		
14.0 ± 2.0 (9.5–18.9)	40 ± 21 (6–89)	11.9 ± 3.3 (4.6–16.6)
N = 73		N = 70
**Female**		
14.0 ± 2.1 (9.5–18.9)	40 ± 21 (10–89)	11.9 ± 3.4 (4.6–17.5)
N = 68		N = 66
Male		
14.3 ± 1.0 (12.8–15.4)	26 ± 15 (6–76)	12.6 ± 2.1 (9.7–14.7)
N = 5		N = 4

**Table 2:** Clinical and demographic characteristics of the participants in the first replication cohort.

AIS patients		Healthy controls	CARTaGENE subjects
Age (years)	Highest Cobb Angle (°)	Age (years)	Age (years)
14.2 ± 2.2 (9.2–19.7)	48 ± 15 (12–85)	13.1 ± 2.8 (5.9–18.3)	NA
N = 96		N = 36	N = 60

**Table 3:** Clinical and demographic characteristics of the participants in the second replication cohort.

Severe AIS patients (≥ 40°)		Moderate AIS patients (< 40°)		Healthy controls
Age (years)	Highest Cobb Angle (°)	Age (years)	Highest Cobb Angle (°)	Age (years)
**All**				
15.8 ± 2.1 (10.9–21.5)	52.9 ± 9.1 (40–74)	16.8 ± 0.96 (15.2–20.1)	19.7 ± 6.3 (10–33)	12.5 ± 3.2 (3.2–18.3)
N = 111		N = 147		N = 143
**Female**				
15.6 ± 1.9 (10.9–21.2)	54.1 ± 9.4 (43–74)	16.8 ± 0.99 (15.2–20.1)	20.1 ± 6.1 (10–33)	12.5 ± 3.3 (4.3–18.3)
N = 94		N = 120		N = 67
**Male**				
16.8 ± 2.5 (11.9–21.5)	47.2 ± 5.4 (40–54)	16.8 ± 0.8 (15.6–19.2)	18 ± 7.2 (10–29)	12.5 ± 3.2 (3.2–17.6)
N = 17		N = 27		N = 76

### Whole-exome sequencing (WES)

We performed WES using our discovery cohort, followed by variant annotation and filtering to identify rare variants contributing to AIS. To enhance statistical power, we examined genes harboring an overall excess of rare variants in the discovery patient cohort. We performed a collapsing gene burden test, in which we compared the enrichment of rare variants per gene in patients versus controls. To define rare variants, we applied a minor allele frequency (MAF) < 1% as an initial cutoff, and MAF < 0.5% as a more stringent cutoff according to the 1000 Genomes Project European ancestry (EUR) and the Exome Sequencing Project European ancestry (ESP-EA). Only 8150 genes harbored at least one such rare variant among all case and control samples. Therefore, we set a statistical significance threshold at 0.05/8150 = 6 × 10^−6^. Based on our results, none of the 8150 genes met the p-value threshold. We therefore selected the top 24 genes with the strongest statistical scores, for follow-up validation in an independent replication cohort (Table 4). To selection the 24 candidate genes, we took into consideration both the p-values and the absolutes numbers of patients and controls who carried the rare variants.

**Table 4:** Genes selected from the discovery cohort with SNPs of MAF < 1%.

Gene	Cases with rare non-synonymous SNPs	Controls with rare non-synonymous SNPs	Whole-exome uncorrected Fisher exact two-tailed *p* value
*GLP1R*	10	0	0.001
*DMRT3*	10	1	0.009
*ITGA8*	7	0	0.014
*A1CF*	7	0	0.014
*GPR179*	14	4	0.022
*FAT3*	12	3	0.027
*CEACAM18*	6	0	0.028
*TTC21A*	6	0	0.028
*NFRKB*	6	0	0.028
*GMPR2*	6	0	0.028
*SLC3A1*	6	0	0.028
*IMMT*	6	0	0.028
*ZNF189*	6	0	0.028
*CD1B*	10	2	0.031
*SEC16A*	10	2	0.031
*CCDC50*	8	1	0.034
*SLC22A16*	12	4	0.062
*R3HCC1L*	7	1	0.063
*IL16*	7	1	0.063
*HPS4*	6	1	0.116
*DPEP3*	6	1	0.116
*LY75*	6	1	0.116
*TENM3*	6	1	0.116
*ITGA4*	4	0	0.120

### Targeted sequencing of the selected 24 genes in a first replication cohort

**Figure 1:**
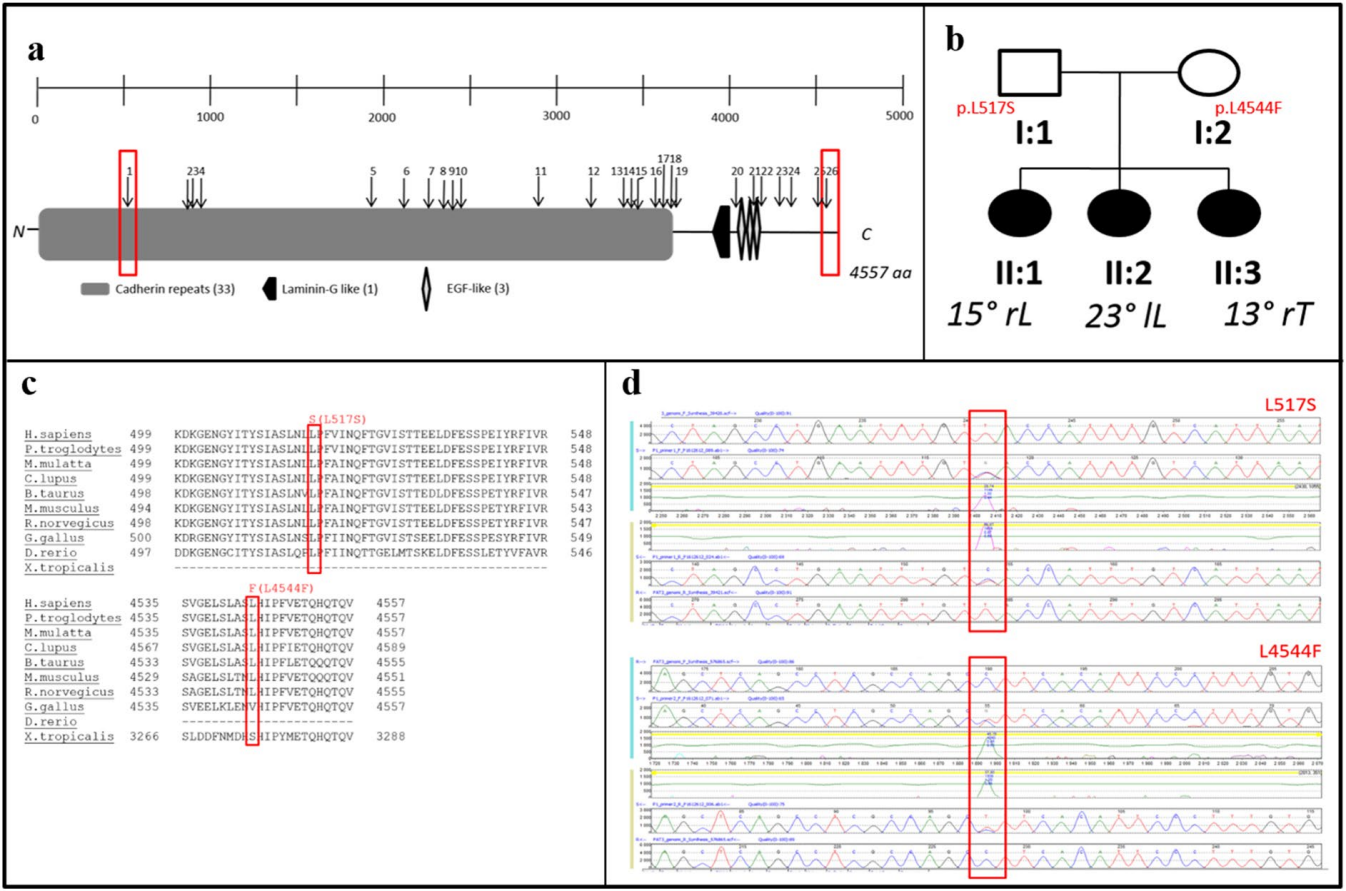
Multiplex AIS family. One family in our cohort (ID1581) consisted of three affected sisters and two unaffected parents. (**a**) FAT3 protein organization as annotated by NCBI is 4557 amino acids long and includes multiple functional homology domains. The positions of the 26 rare variants identified in our study among the AIS cases are labelled from 1 to 26 and are indicated by vertical arrows above the protein schema. The location of two heterozygous mutations present in this multiplex AIS family are indicated by the red boxes. (**b**) A simplified pedigree and segregation of the *FAT3* mutations. (**c**) A sequence alignment with different species showing that both mutations affect an invariantly conserved amino acid sequences in FAT3 orthologues. (**d**) Sequence chromatograms showing those heterozygous mutations.

The first replication cohort was chosen to be more homogeneous; all cases were severely affected females. By comparison, in our initial discovery cohort, 93% of AIS patients were females and only 68% were severe cases. This replication cohort includes 96 female patients and 96 female controls. The exons of the 24 genes were sequenced in the 192-replication samples using a custom capture library. After calling and annotating variants, we first removed poor quality calls, variants with a MAF ≥ 1% (according to the 1000 Genomes Project EUR and the gnomAD whole-genome and whole-exome databases), and synonymous variants. We included near-intronic variants that might affect efficiency of RNA splicing. Similarly, we employed the collapsing gene burden test. Of the 24 genes, only one gene, *FAT3*, continued to show an enrichment of rare, potentially protein-altering variants in patients versus controls. Specifically, there were 21 rare variants in *FAT3*, compared to 11 in controls (uncorrected *p* value = 0.04, Fisher-exact one-tailed test) (Table 5). We suggest that a one-tailed-test is appropriate since the primary ascertainment was for AIS cases, and rare variants in our cohorts would not realistically be expected to be protective. The *p* value of 0.04 is before correcting for multiple genes in the replication thus is not formally statistically significant although it is highly suggestive. Importantly, we explored other models, such as using a 2% MAF or even 5% MAF threshold instead of 1%, or filtering to retain variants with a REVEL pathogenicity score above 0.3 (the value for which REVEL specificity and sensitivity are approximately equal). The total number of variants changed with each of these alternative definitions, however *FAT3* continued to be the only gene with a significant excess of cases versus controls with variants in all these tests (see Supplementary Tables S2 and S3 for altered MAF thresholds). Including synonymous variants however eliminated the case/control difference in *FAT3* as well (data not shown). The protein-altering variants in *FAT3* in both discovery and replication cohorts were distributed across much of the protein encoded by *FAT3* (Fig. 1a).

**Table 5:** Statistical analysis for all selected genes in the replication cohort with potentially protein-altering SNPs with MAF ≤ 1%.

Gene	Variants in cases	Variants in controls	Fisher exact one-tailed *p* value
A1CF	5	5	–
CCDC50	2	1	–
CD1B	1	3	–
CEACAM18	3	1	–
DMRT3	4	3	–
DPEP3	6	5	–
FAT3	21	11	**0.04**
GLP1R	2	2	–
GPR179	12	12	1
HPS4	7	2	–
IL16	7	9	0.79
IMMT	0	5	–
ITGA4	3	6	–
ITGA8	6	3	–
LY75	9	9	1
NFRKB	4	7	–
R3HCC1L	10	7	0.62
SEC16A	8	8	1
SLC22A16	2	2	–
SLC3A1	2	5	–
TENM3	6	5	–
TTC21A	1	5	–
ZNF189	0	1	–

Significant values are in [bold].

### Whole-exome sequencing of independent AIS family

Independently of the AIS case/control cohort, we ascertained a rare multiplex family in which three sisters were affected with AIS while the parents were unaffected (Fig. 1b). Consistent with the case–control WES and targeted gene sequencing analyzes, we restricted the analysis to rare (MAF ≤ 1%), potential protein-altering SNPs or small insertions and deletions (indels). We analyzed the family WES data with different inheritance models, given the unaffected status of both parents. First, we considered a de novo mutation model in which the three sisters would share a heterozygous variant absent in the parents. Second, we considered a recessive model; either homozygous variant in the three sisters (which is heterozygous in the parents) or compound heterozygous for which the three sisters have two heterozygous variants in the same gene, each coming from one parent. Our results showed that no genes were consistent with the de novo or homozygous recessive models. However, the presence of compound heterozygous variants in *FAT3* were found. Of note, the selection of candidate genes, which included the same *FAT3* gene, from unbiased WES of the case–control cohort was done before we performed the family study. The two *FAT3* variants found in the multiplex family are non-synonymous: p.L517S (rs139595720) and p.L4544F (rs187159256) (Fig. 1c). Both variants were confirmed by Sanger sequencing of DNA from all members of the family (Fig. 1d). The first variant was also present in four cases and one control in the replication cohort, and the second variant was present in one case in the discovery cohort.

### Validation of *FAT3* gene structure and identification of a novel unannotated exon

The gene model for *FAT3* used by RefSeq appears to be supported mainly by long individual rodent cDNA clones in the NCBI database, whereas there are only fragmentary human cDNA clones documented in the public genome browsers. Therefore, to confirm the human *FAT3* gene structure, we analyzed our in-house brain RNA-Seq data and whole-genome bisulfite sequencing (WGBS) data for one individual. Our results were consistent with the RefSeq gene model (NM_001008781.2) with two exceptions. Just upstream of the 3’ terminal exon we found evidence for two alternative exons which were either included or excluded together in various RNA-Seq reads. The two exons are also annotated by the GENCODE project website (version 24). In addition, we identified a previously uncharacterized exon located 125 kb upstream of the first annotated exon, supported by multiple individual reads splicing this sequence to the second (but first protein-coding) exon (Supplementary Fig. S1). This novel exon lies in a hypomethylated CpG island, a feature that is characteristic of active promoters (Supplementary Fig. S2). Because the 5′-most exon annotated by GENCODE (exon 2 of our gene model) begins precisely at the splice acceptor junction, we suspect that the GENCODE raw data probably included exon 1 in some junction reads, which were not aligned to the genome across exon 1 due to the very long first intron. We also profiled *FAT3* expression using GTExTranscriptome Portal and observed a strong enrichment in brain and artery tissues (Supplementary Fig. S3; note that in the course of preparation of this manuscript, an additional RefSeq annotation for *FAT3* has appeared, NM_001367949.1, which included an additional exon in the CpG island).

### Sanger sequencing of exons 25 and 26 of *FAT3*

The alternative exons 25 and 26 were not captured in the replication capture sequencing because they are not annotated by RefSeq. Hence, we performed direct Sanger sequencing for these two exons in 72 cases of the first replication cohort (DNA was not available for the rest of the cases). No rare, potentially protein-altering variants were observed among the sequenced cases for either of these two (very small) exons (data not shown).

### Consequences of the *FAT3* rare variants

We identified in AIS patients 26 non-synonymous SNVs (25 previously reported in public databases and 1 novel) in the *FAT3* gene (Table 6). Prediction of the functional consequences of the non-synonymous SNVs was performed using three different algorithms including SFIT, PolyPhen-2 and MutationTaster2. Of note, two variants were predicted as likely pathogenic by all three algorithms, 13 variants as likely pathogenic by two of the three algorithms, and one variant is a frame shift mutation (Table 6). To test whether these rare variants affect the expression of *FAT3*, we performed qPCR expression analysis using RNA extracted from primary osteoblasts obtained from seven scoliotic patients who had rare variants in *FAT3* from the discovery and replication cohorts, and seven controls (trauma patients who did not have scoliosis and from whom we could extract osteoblasts). No statistically significant difference in averaged *FAT3* expression was observed between the two groups (Supplementary Fig. S4). We did a second validation step using another independent replication cohort (replication cohort 2, Table 3) using well characterized AIS patients having reached skeletal maturity, to genotype nine *FAT3* gene variants including the two variants previously identified in the multiplex family: p.L517S (rs139595720) and p.L4544F (rs187159256). Interestingly, two *FAT3* variants rs139595720 (genotype A/G) and rs80293525 (genotype C/T) were enriched in scoliosis ≥ 40° (4.5% and 2.7% respectively) compared to < 40° (1.4% and 0.7%) and controls (1.2% and 0.8%). Whereas the variant rs142403035 (genotype A/G) was associated with less severe spinal deformities with a prevalence of 1.7% in scoliosis cases ≥ 40° compared to 2.1% and 4.4% in < 40° scoliosis cases and controls respectively (Table 7).

**Table 6:** Prediction of *FAT3* variant effects on the function of the protein.

Reference position*	Mutation DNA level (hg19)	Mutation protein level	SNP ID	SIFT^a^	POLYPHEN-2^b^	MUTATION TASTER^c^	REVEL^d^	MAF gnomAD genomes^e^
1	chr11:92086828 T > C	p.L517S	rs139595720	T	D	N	0.303	0.0054
2	chr11:92087676A > T	p.N800Y	rs188857169	D	D	D	**0.543**	0.00S073
3	chr11:92087959G > A	p.R894Q	rs80293525	T	D	D	**0.488**	0.0055
4	chr11:92088151 T > C	p.L958P	rs76869520	D	D	D	**0.821**	0.00038
5	chr11:92532013A > G	p.N1945S	rs749177833	T	B	D	0.074	0.000036
6	chr11:92532651A > G	p.I2158V	rs780333216	T	B	N	0.073	0.000021
7	chr11:92533254C > T	p.H2359Y	rs80046666	T	B	N	0.096	0.0084
8	chr11:92533405G > A	p.R2409Q	rs538822881	T	D	D	0.213	0.000064
9	chr11:92533555A > G	p.Q2459R	rs118056487	T	D	D	0.298	0.0025
10	chr11:92533558G > A	p.R2460Q	rs200944979	T	D	D	0.205	0.00073
11	chr11:92534695 T > C	p.I2839T	rs200241295	T	B	N	0.038	0.00048
12	chr11:92565003C > A	p.P3233T	rs752644378	T	D	D	0.282	0.000029
13	chr11:92569867C > T	p.R3408W	rs200404766	D	D	N	0.173	0.0018
14	chr11:92570856G > T	p.A3418S	rs201449521	D	B	D	**0.721**	0.0021
15	chr11:92573811CT > C	p.S3485fs	novel	–	–	–	–	–
16	chr11:92577352G > A	p.A3607T	rs200032318	T	D	D	**0.463**	0.0022
17	chr11:92577469C > A	p.Q3646K	rs555950318	T	B	D	0.165	0.000012
18	chr11:92577590G > A	p.R3686H	rs138237129	T	B	N	0.134	0.00083
19	chr11:92577659G > T	p.S3709I	rs75081660	T	B	D	0.053	0.0072
20	chr11:92613978G > A	p.R4070Q	rs201379307	T	B	D	**0.383**	0.0010
21	chr11:92616191C > T	p.T4190M	rs186899262	T	D	D	0.350	0.0006
22	chr11:92616217G > A	p.A4199T	rs201053443	T	D	D	0.335	0.00026
23	chr11:92620226A > G	p.N4333S	rs765678336	T	D	D	0.198	0.000008
24	chr11:92623798G > A	p.G4398D	rs142403035	D	P	D	**0.743**	0.0011
25	chr11:92624166 T > C	p.C4521R	rs1486678306	D	B	D	0.317	0.000008
26	chr11:92624235C > T	p.L4544F	rs187159256	D	B	N	0.223	0.0035

^a^SIFT: D, damaging, T, tolerated.

^b^POLYPHEN-2: D, probably damaging, P, possibly damaging, B, benign.

^c^MUTATION TASTER: D, disease causing, N, polymorphism.

^d^REVEL: score from 0–1, less to more pathogenic, sensitivity equal to specificity at 0.380.

^e^In a few cases MAF is from gnomAD Exomes, normally essentially identical to genomes.

*Position of each variant of FAT3 protein is illustrated in Fig. 1a.

Significant values are in bold.

**Table 7:** Genotyping of selected nine variants in *FAT3* gene in second replication AIS cohort.

SNP number	Healthy control	Severe AIS (≥ 40°)	Moderate AIS (< 40°)
**rs139595720**			
N	129	111	142
AA	127 (98.4%)	106 (95.5%)	140 (98.6)
AG	2 (1.6%)	5 (4.5%)	2 (1.4%)
**rs188857169**			
n	130	115	147
TT	130 (100%)	114 (99.1%)	147 (100%)
AT	0 (0%)	1 (0.9%)	0 (0%)
**rs80293525**			
n	129	112	145
CC	128 (99.2%)	109 (97.3%)	144 (99.3%)
CT	1 (0.8%)	3 (2.7%)	1 (0.7%)
**rs76869520**			
n	129	110	143
TT	129 (100%)	109 (99.1%)	142 (99.2%)
CT	0 (0%)	1 (0.9%)	1 (0.7%)
**rs200944979**			
n	141	117	149
CC	139 (98.6%)	116 (99.1%)	149 (100%)
CT	2 (1.4%)	1 (0.9%)	0 (0%)
**rs186899262**			
n	80	80	114
CC	80 (100%)	80 (100%)	114 (100%)
**rs201053443**			
n	130	114	146
CC	130 (100%)	113 (99.1%)	146 (100%)
CT	0 (0%)	1 (0.9%)	0 (0%)
**rs142403035**			
n	136	116	148
GG	130 (95.6%)	114 (98.3%)	145 (97.9%)
AG	6 (4.4%)	2 (1.7%)	3 (2.1%)
**rs187159256**			
n	128	109	143
CC	128 (100%)	108 (99.1%)	142 (99.3%)
CT	0 (0%)	1 (0.9%)	1 (0.7%)

## Discussion

Using WES with a combined two-stage, case/control and multiplex family approach, we discovered a new association between the *FAT3* gene and AIS. Although the cohort-based gene burden test did not achieve full statistical significance after correcting for multiple gene testing, the observation of compound heterozygous variants in *FAT3* in all three affected siblings in an independently ascertained multiplex AIS family (itself a very unusual occurrence), strongly supports the identification of *FAT3* as an interesting candidate gene in AIS. The failure to achieve full statistical significance is likely due to the size limitation of our cohorts. It should be noted that population stratification or bias between cases and controls is unlikely since both were similarly obtained from the general Quebec school population.

Most other studies of AIS genetics have looked for individual rare variants in families^23^, rather than collapsing these variants by genes. *POC5* and *HSPG2* were initially identified from such familial studies, and only then were further investigated in independent cohorts^21,22^. Only two studies employed an approach similar to ours, looking at rare variant burden at the gene level. Buchan et al.^20^, with a two-stage approach beginning with WES of 91 severe AIS cases and a collapsing gene burden test^25^, followed by targeted gene resequencing in a second, much larger cohort. As in our study, no single gene achieved genome-wide significance, but the gene with the smallest *p* value, *FBN1*, was pursued in a replication cohort similar to our approach and replicated together with the related gene *FBN2*. In the second study, Haller et al., analyzed exome sequence data of 391 severe AIS cases and 843 controls. Again, in a genome-wide gene burden test no individual gene achieved statistical significance, therefore, they further collapsed genes according to gene ontology pathways and observed excess variation among genes implicated in the extracellular matrix, particularly collagen genes^24^. No collagen or fibrillin genes were among the 24 candidates in our replication cohort. Exome-wide genetic analysis are generally vulnerable to biases. However, the use of custom exon capture kits resulted in very high coverage of target gene exons, limiting false positive and negative errors.

AIS is a highly heterogeneous disease in terms of both phenotype and etiology, therefore finding a common genetic background in isolated cases is challenging. Several of the individual rare variants we observed in our case cohort were recurrent, suggestive of at least a modest founder effect. This is consistent with the elevated incidence of scoliosis in Quebec, given that our cohort was almost completely of French-Canadian ancestry. Nonetheless, there were a relatively large number of different rare variants in our cases versus matched controls. Our identification of *FAT3* as a potential candidate gene with this strategy may also have depended on a very homogeneous phenotype definition in terms of sex and severity. It is also worth noting that our controls are not random population controls, but are effectively discordant since they are of individuals whose physical exam and the lack of family antecedents excludes a diagnosis of AIS or related spinal disorders. It would be interesting to revisit the total variant data sets from the previous population studies^20,24^ with respect to *FAT3*; however, those data are not available to us. More generally, our results indicate that two-stage approaches for rare variant detection in common complex diseases can yield good gene candidates for further study, even without additional criteria relying on previously known biology of the disease.

Interestingly, *FAT3* is near another gene *MTNR1B* melatonin receptor 1B, in which a polymorphism has been associated with AIS^26^. The SNP in question, rs4753426, lies slightly proximal to the 5′ end of *MTNR1B*, and about 72 kb distal to the 3′ end of *FAT3*. We speculate that the observed association may be functionally related to *FAT3* rather than *MTNR1B* function, especially as the association is strongest in Asian populations where there is typically more extended linkage disequilibrium.

*FAT3* is a member of the FAT gene family comprised of *FAT1*, *FAT2*, *FAT3* and *FAT4*, all of which are members of the cadherin super family homologous to the *Drosophila* gene *Fat*^27^ regulating planar cell polarity (PCP) in the Drosophila wing^28^. Members of the FAT cadherin subfamily have conserved structures from flies to vertebrates^29^. *FAT3* contains multiple repeats of a cadherin repeat domain (involved in Ca^+2^ binding), a single laminin G domain and three EGF-like Ca^+2^ binding domains. The rare non-synonymous variants that we observed in our discovery and replication cohorts are distributed across much of the protein, including some in these conserved domain regions (Fig. 1A). Mutations in each of the FAT genes has been reported in many types of cancers including early T-cell precursor acute lymphoblastic leukemia^30^, ovarian^31^, and pancreatic^32^. It is presumed that they all represent somatic, not inherited mutations, although it is difficult to confirm this among the various sequencing studies. There is no particular known co-morbidity between AIS and such cancers. More interestingly, multiple rare variants in *FAT3* were reported in families affected by the developmental disorder Hirschsprung disease^33^; two of the reported variants are present in our first stage discovery case cohort. As far as we know, there is no phenotypic component related to Hirschsprung in our cohorts. Although Hirschsprung disease is not obviously developmentally related to scoliosis, there are scattered reports in the literature of co-morbidity of these conditions^34–36^. Given the wide variety of developmental functions ascribed to the *FAT* genes, genetic associations of either common or rare variants to multiple complex disorders are plausible.

Somatic mutations in *FAT3* affect cell adhesion and interaction mechanisms, beside affecting the Wnt pathway^30^. Members of the FAT family proteins work synergistically and antagonistically to affect many aspects of tissue morphogenesis^37^. It has been shown that FAT3 and FAT4 act synergistically during fusion of the vertebral arches^37^ through conserved interactions with components of planar polarity pathways. *Fat3* knockout mice have planar polarity defects^37^. A recent study demonstrated that a targeted mutation in the zebrafish *D. rerio* ptk7 gene, whose encoded protein functions in cell communication, leads to both congenital and idiopathic scoliosis according to the timing of gene loss of function. Furthermore, mutation of the gene led to the disruption of both planar cell polarity (PCP) and Wnt/ß-catenin signaling, consistent with the contribution of these pathways to the disease^38^. The PCP pathways play an important role in regulating the polarity and behavior of different cells in different tissues^39^. Le Pabic et al.^39^ suggested that PCP might be involved in skeletal morphogenesis as well. They proposed a model whereby FAT3 coordinates the polarity and differentiation of chondrocytes affecting skeletal morphology. *FAT3* is highly expressed in the nervous system and affects the neuronal morphology^40^, beside its expression in the intervertebral discs^41^, vertebral bone and other bone cells. As mentioned, two *FAT3* variants (rs139595720 and rs187159256) are associated with scoliosis severity as demonstrated by the higher frequencies of the heterozygous genotypes in severe scoliosis (≥ 40°) compared to moderate scoliosis (< 40°) and healthy controls. According to the POLYPHEN-2 analysis, the variant rs139595720 (p.L517S) is probably damaging and await additional experiments to confirm.

We directly compared the *FAT3* gene expression levels in bone cells in a subset of our patients harboring rare variants in the gene to a group of controls lacking such variants. However, these rare variants in *FAT3* appeared to have no statistically significant effect on expression of the gene at least in this cell type. Somewhat unexpectedly, the statistical support for association of rare variants in *FAT3* with AIS was stronger when synonymous variants were included. It has been shown that synonymous variants can affect mRNA splicing^42^, mRNA stability and protein expression^43^, and even in one case protein conformation and function^44^. We were not able to explore this directly due to lack of available biological materials from the particular cases in our cohort harboring such rare synonymous variants. However, the rare non-synonymous variants in our cases were not obviously clustered near exonic splice junctions.

In summary, our results implicate *FAT3* as an interesting gene candidate contributing to either the occurrence or severity (or both) of AIS.

## Materials and methods

### Study populations

All patients with AIS were examined by orthopedic surgeons from the three pediatric centers participating in this study. A diagnosis of AIS required both history and physical examination with a minimum curvature in the coronal plane of 10°, showed by a standing postero-anterior spinal radiograph, by the Cobb method with vertebral rotation and without any known congenital or genetic disorder. Healthy children were recruited from schools in the Montreal area, and examined by a participating orthopedic surgeon. This study was approved by the institutional review boards of Sainte-Justine University Hospital, The Montreal Children’s Hospital, The Shriners Hospital for Children, and McGill University, as well as The Affluent and Montreal English School Boards. Written informed consents were given by parents or legal guardians and assents were given all minors. All methods were carried out in accordance with relevant guidelines and regulations.

### Discovery AIS cohort

We selected 73 unrelated AIS cases and 70 sex- and age-matched healthy controls. All participants were of French-Canadian ancestry. Fifty of the cases were severe (Cobb angle ≥ 40°) and 23 were moderate (Cobb angle < 40°) (Table 1). Healthy controls were all scanned for spinal curvatures using a scoliometer and forward bending-test by an orthopedist surgeon. Moreover, healthy individuals with a family history of scoliosis were excluded.

### Replication AIS cohort 1

Ninety-six patients of French-Canadian origin were selected for the first replication study, unrelated to each other or to the cases in the discovery cohort. Since 93% of the initial cohort were females and 68% were severe cases, the second cohort were chosen to be all females and severely affected. Thirty-six healthy French-Canadian females were recruited from Montreal schools, and an additional 60 French-Canadian females from the CARTAGENE project^45,46^ (Table 2).

### Replication AIS cohort 2

Two-hundred fifty-eight patients of French–Canadian origin were selected for the second replication study, unrelated to each other or to the cases in the discovery and first replication cohort. One hundred forty-three healthy controls were recruited from Montreal’s schools (Table 3). All scoliosis patients reached their skeletal maturity and were divided as severe scoliosis (≥ 40°) (N = 111) or moderate scoliosis (< 40°) (N = 147).

### French–Canadian multiplex family

A rare multiplex French-Canadian family with three affected sisters and healthy parents was ascertained and analyzed by WES analysis. The proband was diagnosed with AIS at the age of 13 years old with a right lumbar curve and a Cobb angle measuring 15°. Her first sister was diagnosed with AIS with a left lumbar curve measuring 23° and the second sister was also diagnosed with AIS with right thoracic curve measuring 13°.

### DNA extraction

Blood was obtained by standard venipuncture. Genomic DNA was extracted from peripheral leukocytes using PureLink genomic DNA kit (Thermo Fisher Scientific, Waltham, Massachusetts, USA).

### Whole-exome sequencing of discovery cohort

Exome capture was performed using Agilent SureSelect^XT^ Human All Exon 50 Mb v3 according to the manufacturer’s recommendations. Sequencing was done using Applied Biosystems’ SOLiD 5500xl at the Sainte-Justine University Hospital genomic platform. The average coverage of targeted sites was approximately 100X (Supplemental Information).

### Targeted deep sequencing of selected genes in a replication French–Canadian AIS cohort

Twenty-four genes were chosen for resequencing in a second French–Canadian cohort. Enrichment of coding exons of these genes was done using Roche NimbleGen’s EZ Choice custom baits, with bar code multiplexing of 96 samples per lane of sequencing. Sequencing was done on an Illumina HiSeq 2000 and performed at the McGill University and Genome Quebec Innovation Centre (MUGQIC). The average coverage of targeted sites was approximately 400× (Supplemental Information). Although there was some scatter, in general there were equivalent numbers of variant calls in all genes in the control category of total common plus rare exonic, plus near intronic variants (Supplementary Table S1).

### Whole-exome sequencing of a French–Canadian family

Exome capture for the multiplex family was performed using Agilent SureSelect Human All Exon 50 Mb v3 according to the manufacturer’s recommendations. Sequencing was done on an Illumina HiSeq2500 at the Sainte-Justine University Hospital genomic platform (Supplemental Information).

### Sequencing data processing

The details of our bioinformatics analysis, pipeline and subsequent variant filters are shown in the Supplemental Information. Only protein coding, and near intronic regions were analyzed. Our analysis included SNPs, and small indels. The SIFT (Sorting Intolerant from Tolerant)^47^, PolyPhen-2 (Polymorphism Phenotyping v2)^48^ and MutationTaster2^49^ algorithms were used to predict possible impact of amino acid substitutions on the structure and function of a human FAT3 protein in AIS patients harboring different *FAT3* gene variants.

### Sanger sequencing

Sanger sequencing was performed at the Genome Quebec Innovation Centre at McGill University. Primers were designed using the program Primer3. Sanger sequence chromatograms were analyzed using Mutation Surveyor. Exons 25 and 26 of *FAT3* were not initially sequenced in the replication cohort because the custom baits used to capture the selected genes for sequencing were designed according to the RefSeq gene model, which did not include those two alternative exons. Hence, we performed Sanger sequencing for the two additional exons in 72 patients of the replication cohort. DNA of the other patients was not available. Numbering of variants in *FAT3* is based on NCBI reference sequence entries NM_001008781.2 and NP_001008781.2.

### Genotyping of SNPs in the *FAT3* gene

Genomic DNA samples were derived from the peripheral blood of the subjects of the second replication cohort using PureLink Genomic DNA kit. Nine SNPs were genotyped in the *FAT3* gene (Table 7). Multiplex PCR of the nine SNPs was performed at McGill University and Genome Quebec Innovation using standard procedures with 20 ng of template genomic DNA and HotStarTaq DNA polymerase enzyme. PCR reactions were run on the QIAxcel (Qiagen) to assess the amplification, followed by the single base extension using iPlex Thermo Sequenase. Genotypes were determined by MALDI-TOF mass-spectrometry and data were analyzed using Mass ARRAY Typer Analyser software.

### Statistical analyses

In both phases of case/control analyses, we employed a collapsing gene burden test for significance testing, under the assumption that all rare, potentially protein-altering variants act in the same phenotypic direction with the same magnitude, independent of specific allele frequencies. In the few instances where an individual carried two rare variants in the same gene, these were counted as independent events generating a gene-allele burden count rather than a case count. In the first, discovery WES phase, chi-square p-values were calculated to compare the accumulation of rare variants (MAF < 0.01) in genes throughout the exome in patients versus controls, assuming a significance threshold of *p* = 6 × 10^−6^ (0.05/8150), based on the number of genes harboring at least one rare variant among either cases or controls in the WES data set. In the targeted gene phase (24 selected genes), Fisher’s exact test was used to calculate one-tailed *p* values for comparisons between patients and controls using GraphPad (https://www.graphpad.com/data-analysis-resource-center/#quickcalcs), with the statistical significance threshold corrected for the number of genes having a minimum number of rare variants as described under the “Results” section. When used, REVEL scores were used based on the pre-computed database; however, protein-truncating variants (stop gains, frameshift insertion/deletions), which are normally not assigned REVEL scores, were given a score of 1 for maximal predicted pathogenicity. REVEL scores are equally not assigned for intronic variants regardless whether they might affect splicing efficiency.

### Validation of *FAT3* gene structure

The gene model for *FAT3* used by RefSeq does not appear to be supported by long individual human cDNA clones, and seems to be based on homology to several long rodent cDNAs. Therefore, to confirm the gene structure, we analyzed in-house brain RNA-Seq data and WGBS data from an unrelated individual not part of our cohorts, as well as from GENCODE public annotations. We also profiled *FAT3* expression using GTExTranscriptome Portal (http://www.gtexportal.org/home/gene/FAT3).

### Cell culture and RNA extraction

Primary osteoblasts were derived from bone specimens obtained intraoperatively from AIS and non-scoliotic trauma cases. Briefly, cells were grown in 10 cm^2^ culture dishes with Alpha Modification of Eagle’s Medium (αMEM) containing 10% fetal bovine serum (FBS) and 1% antibiotic/antimycotic at 37 °C and 5% CO_2._ Cells were grown until they reached confluence. Then, the cells were washed with phosphate-buffered saline (PBS 1×) twice and treated with 1 ml TRIzol, lysed and transferred to 1.5 ml tube and stored at − 80 °C. RNA was extracted using TRIzol, following the manufacturer’s instructions.

## Quantitative RT-polymerase chain reaction (qRT-PCR)

Expression analyses by qRT-PCR were done in triplicate using *GAPDH* and *PPIA* (Peptidylprolyl isomerase A) as normalizing housekeeping genes (Supplemental Information).

## Supplementary Information

Supplementary Information.

## Data Availability

The known variant datasets analyzed in the current study are available in the dbSNP and gnomAD repositories. All other data generated cannot be deposited publicly as it is prohibited by our institutional review board. The corresponding author may be contacted to gain access to this data. Researches wishing to access the data will have to submit their own study’s approved protocol and consent forms for review.
