# Supplementary material for: Identification of FAT3 as a new candidate gene for adolescent idiopathic scoliosis
Source: Sci Rep. 2022 Jul 19;12:12298. doi: 10.1038/s41598-022-16620-6 (PMC9296578; doi:10.1038/s41598-022-16620-6)
Supplement: Supplementary file 1 — Supplementary Information. [file 41598_2022_16620_MOESM1_ESM.docx]

**Identification of FAT3 as a new candidate gene for adolescent idiopathic scoliosis**

Dina Nada^1,2^, Cédric Julien^1,3^, Simon Papillon-Cavanagh^4^, Jacek Majewski^4^, Mohamed Elbakry^1,5^, Wesam Elremaly^1,6^, Mark E. Samuels^7,8^, Alain Moreau^1,6,9, *^

^1^Viscogliosi Laboratory in Molecular Genetics of Musculoskeletal Diseases, Sainte-Justine University Hospital Research Center, Montreal, QC, Canada;

^2^Pharmacology & Biochemistry Department, Faculty of Pharmacy, The British University in Egypt, Cairo, Egypt;

^3^Injury Repair Recovery Program, McGill University Health Center Research Institute, Montreal, QC, Canada;

^4^Department of Human Genetics, McGill University, Montreal, QC, Canada;

^5^Biochemistry Division, Chemistry Department, Faculty of Science, Tanta University, Tanta, Egypt;

^6^Department of Biochemistry and Molecular Medicine, Faculty of Medicine, Université de Montréal, Montreal, QC, Canada;

^7^Sainte-Justine University Hospital Research Center, Montreal, QC, Canada;

^8^Department of Medicine, Faculty of Medicine, Université de Montréal, Montreal, QC, Canada;

^9^Department of Stomatology, Faculty of Dentistry, Université de Montréal, Montreal, QC, Canada.

* Corresponding Author: Alain Moreau PhD, Viscogliosi Laboratory in Molecular Genetics of Musculoskeletal Diseases, Sainte-Justine University Hospital Research Center (room 2.17.027), 3175 Chemin de la Côte-Ste-Catherine, Montreal, H3T 1C5, QC, Canada;

E-mail: [alain.moreau.hsj@ssss.gouv.qc.ca](mailto:alain.moreau.hsj@ssss.gouv.qc.ca) or [alain.moreau@umontreal.ca](mailto:alain.moreau@umontreal.ca)

**SUPPLEMENTAL INFORMATION**

**SOLiD 5500xl WES of the discovery cohort.** Libraries were constructed using a modified version of the Fragment Library Preparation 5500 Series SOLiD Systems User Guide. Genomic DNAwas fragmented with a Covaris S2 System, then using a Bioanalyzer (Agilent) for quantification, 3 μg was used for library construction. Truncated adaptors were utilized to minimize nonspecific capture during SureSelectin-solution hybridization. The P1-T and barcode-T-0XX adaptors were replaced by:

Tr5500P1

5′- CCTCTCTATGGGCAGTCGGTGA*T -3′

3’- C*C*GGAGAGATACCCGTCAGCCACT -5’

Tr5500IA

5′- CGCCTTGGCCGTACAGC -3′

3’ T*GCGGAACCGGCATGTCG*T*C -5’

* Phosphorothioate bond

In addition, Library PCR Primer 1 and Library PCR Primer 2 were replaced by SureSelect Pre-Capture Primers provided in the SureSelect AB Barcoding Library Kit (Agilent). Exome capture was performed using Agilent SureSelectXT Human All Exon 50 Mb v3 according to the manufacturer’s recommendations. Final libraries were quantified using the SOLiD Library TaqMan Quantitation Kit. Standard steps were taken thereafter to create enriched, templated beads for the SOLiD 5500xl system. Pools of 8 libraries were loaded on each flowchip (6 lanes). Sequencing was performed in paired-end 50 bases in forward and 25 bases in reverse.

# HiSeq2500 WES for the French Canadian family. Genomic DNA was quantified using the QuantiFluor dsDNA System (Promega) and 3µg each was used as input. Libraries were constructed using the SureSelect^XT^ Target Enrichment System for Illumina Paired-End Multiplexed Sequencing Library protocol and the SureSelect^XT^Human All Exon 50 Mb v5 capture kit (Agilent). Final libraries were qualified using a Bioanalyzer and quantified using the KAPA Library Quantification kit for Illumina. The clustering was done on a Illumina cBot using 16pM of pooled libraries. Pools of 4 librairies were loaded on each lane of a High Output flowcell (8 lanes). Sequencing was performed on a HiSeq2500 for 125 cycles in paired-end using HCS 2.2.38 and RTA 1.18.61.

# HiSeq2000 targeted genes deep sequencing in the replication cohort. Genomic DNA was quantified using the Quant-iT PicoGreen dsDNA Assay Kit (Life Technologies). Libraries were generated robotically using the KAPA HTP Library Preparation Kit Illumina platforms (Kapa Biosystems) as per the manufacturer’s recommendations. TruSeq adapters and PCR primers were purchased from BioO. Libraries were quantified using the Kapa Illumina GA with Revised Primers-SYBR Fast Universal kit (D-Mark). Average size fragment was determined using a LaChip GX (PerkinElmer) instrument. Twenty ng of 48 libraries were pooled together (total of 1000 ng per capture) prior to proceeding with the enrichment of the targeted regions using the Roche Nimblegen EZ Choice custom baits. Captures were performed robotically according the manufacturer’s recommendations.  Final libraries were quantified using the Quant-iT PicoGreen dsDNA Assay Kit and the Kapa Illumina GA with Revised Primers-SYBR Fast Universal kit. Average size fragment was determined using a LaChip GX instrument.The clustering was done on a  Illumina cBot using 11pM of each capture pool (2 captures per lane) and the flowcell was ran on a HiSeq 2000 for 100 cycles in paired-end mode using HCS 2.2.58 and RTA 1.18.63 and using the manufacturer’s instructions.

**Bioinformatic analyses of sequencing data.** The discovery cohort was analysed with the following pipeline:*.xsq files were converted to csfasta and qual file using XSQTools. 5' and 3' reads were filtered independently using SOLiD aware software (<http://bioinformatics.oxfordjournals.org/content/26/6/849.full>) and re-balanced to retain only read pairs i.e. singletons removed. The process was tested to ensure the settings used did not remove too much information, just poor quality reads. Reads were mapped in color space using bfast+bwa-0.7.0a to the reference human genome (hg19) at the library level and then merged by sample. SNPs and INDELs were called with samtools 0.1.19 in batch mode i.e. all sample used during calling. SNPEff 3.3h was used to add genetic variant information and effect prediction (<http://snpeff.sourceforge.net/>). GATK indelRealiger (2.5-2) was used to help resolve indels, Picard MarkDuplicates (1.96) to label PCR duplicates, and GATK base recalibration (2.5-2, SOLiD specific settings) to re-calibrate base qualities due to various sources of systematic technical error. Variants were annotated using Gemini 0.11.1a (<http://gemini.readthedocs.io/en/latest/index.html>).

For quality control validation, Sanger sequencing was performed for more than 100 different variants throughout the exome and results showed consistency in 85% of the genotypes obtained using both sequencing techniques. Based on this, the quality criteria of coverage> 10x, call rate> 90%, map quality > 20. Only variants retained were those with Minor allele frequency < 0.05 in the 1000 Genomes Project, Exome Sequencing Project, ExAC, and dpSNP.

Both the replication French-Canadian IS cohort and the French-Canadian family were analysed using the same pipeline which was slightly different from the discovery cohort. The reads were trimmed and aligned to hg19 using Picard, BWA (0.5.9)(Li and Durbin, 2009)and Samtools (v.0.1.12a) (Li, et al., 2009). Variants were called using Pileup and varFilter commands, followed by filtering to keep SNPs and insertion-deletions of Phred-like quality scores of more than 20 and 50 respectively. Our coverage was approximately 400x for the targeted sequencing and 100x for the five members of the family. Variants were annotated using ANNOVAR (Wang, *et al*., 2010), according to the type of mutation and frequency in the different data bases. We considered both SNPs and small indels. We filtered those which are not in either exonic region or in adjacent intronic sequences including splice site determinants.

**Quantitative Polymerase Chain Reaction (qPCR).** Total RNA was treated with DNase and reverse transcribed using the Maxima First Strand cDNA synthesis kit with ds DNase (Thermo Scientific). Before use, RT samples were diluted 1:5. Gene expression was determined using assays designed with the Universal Probe Library from Roche (www.universalprobelibrary.com). For each qPCR assay, a standard curve was performed to ensure that the efficacy of the assay is between 90% and 110%. QPCR reactions were performed using Perfects QPCR FastMix II (Quanta), 2 µM of each primer and 1 µM of the corresponding UPL probe. The Viia7 qPCR instrument (Life Technologies) was used to detect the amplification level and was programmed with an initial step of 20 sec at 95˚C, followed by 40 cycles of 1 sec at 95˚C and 20 sec at 60˚C.

**REFERENCES**

Li H, Durbin R. Fast and accurate short read alignment with Burrows–Wheeler transform. Bioinformatics 2009;**25**(14):1754-1760.

Li H, Handsaker B, Wysoker A, et al. The sequence alignment/map format and SAMtools. Bioinformatics 2009;**25**(16):2078-2079.

Wang K, Li M, Hakonarson H. ANNOVAR: functional annotation of genetic variants from high-throughput sequencing data. Nucleic acids research 2010; **38**(16):e164-e164.

**SUPPLEMENTARY TABLES**

**Table S1. Counts of total cases or controls in the replication cohort with variants in the replication genes in exonic (including synonymous) plus adjacent intronic sequences (rare plus common)**

| **Gene** | **Count of cases** | **Count of controls** |
| --- | --- | --- |
| A1CF | 13 | 7 |
| CCDC50 | 9 | 11 |
| CD1B | 18 | 14 |
| CEACAM18 | 19 | 19 |
| DMRT3 | 7 | 12 |
| DPEP3 | 9 | 10 |
| FAT3 | 45 | 37 |
| GLP1R | 8 | 10 |
| GMPR2 | 10 | 3 |
| GPR179 | 40 | 48 |
| HPS4 | 15 | 7 |
| IL16 | 13 | 11 |
| IMMT | 11 | 16 |
| ITGA4 | 4 | 11 |
| ITGA8 | 13 | 13 |
| LY75 | 28 | 28 |
| NFRKB | 13 | 12 |
| R3HCC1L | 13 | 17 |
| SEC16A | 57 | 47 |
| SLC22A16 | 16 | 14 |
| SLC3A1 | 6 | 12 |
| TENM3 | 33 | 18 |
| TTC21A | 23 | 43 |
| ZNF189 | 11 | 1 |

**Table S2. Counts of cases or controls with potentially protein-altering (exonic excluding synonymous, plus adjacent intronic) rare variants in the replication genes with MAF<=0.02**

| **Gene** | **Count of cases** | **Count of controls** |
| --- | --- | --- |
| A1CF | 5 | 5 |
| CCDC50 | 2 | 1 |
| CD1B | 9 | 13 |
| CEACAM18 | 3 | 2 |
| DMRT3 | 4 | 3 |
| DPEP3 | 7 | 5 |
| FAT3 | 23 | 13 |
| GLP1R | 6 | 5 |
| GMPR2 | 0 | 0 |
| GPR179 | 24 | 27 |
| HPS4 | 9 | 3 |
| IL16 | 9 | 10 |
| IMMT | 1 | 5 |
| ITGA4 | 3 | 7 |
| ITGA8 | 6 | 3 |
| LY75 | 10 | 10 |
| NFRKB | 4 | 7 |
| R3HCC1L | 10 | 7 |
| SEC16A | 9 | 11 |
| SLC22A16 | 4 | 3 |
| SLC3A1 | 2 | 5 |
| TENM3 | 10 | 8 |
| TTC21A | 8 | 16 |
| ZNF189 | 0 | 1 |

**Table S3. Counts of cases or controls with potentially protein-altering (exonic excluding synonymous, plus adjacent intronic) rare variants in the replication genes with MAF<=0.05**

| **Gene** | **Count of cases** | **Count of controls** |
| --- | --- | --- |
| A1CF | 5 | 5 |
| CCDC50 | 9 | 10 |
| CD1B | 9 | 13 |
| CEACAM18 | 9 | 8 |
| DMRT3 | 5 | 4 |
| DPEP3 | 7 | 5 |
| FAT3 | 31 | 19 |
| GLP1R | 6 | 5 |
| GMPR2 | 0 | 0 |
| GPR179 | 27 | 33 |
| HPS4** | 9 | 3 |
| IL16 | 10 | 11 |
| IMMT | 1 | 5 |
| ITGA4 | 3 | 7 |
| ITGA8 | 10 | 7 |
| LY75 | 12 | 12 |
| NFRKB | 4 | 7 |
| R3HCC1L | 12 | 15 |
| SEC16A | 26 | 24 |
| SLC22A16 | 9 | 8 |
| SLC3A1 | 2 | 5 |
| TENM3 | 10 | 8 |
| TTC21A | 13 | 21 |
| ZNF189 | 0 | 1 |

**SUPPLEMENTARY FIGURES**

**
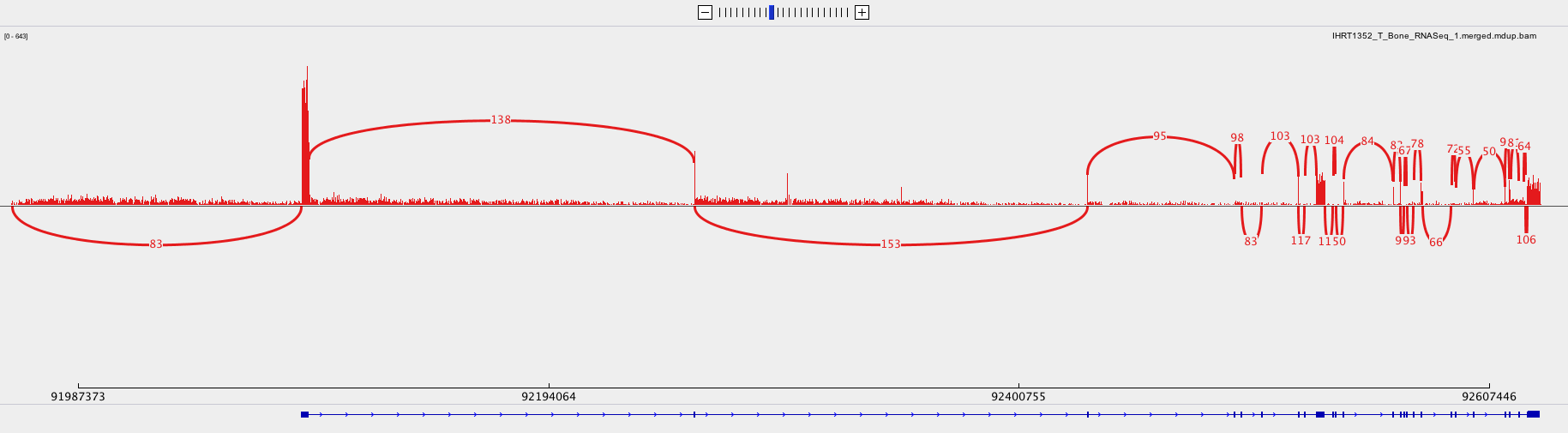
**

**Supplementary Figure S1. Reads (83 reads) supporting the newly identified exon upstream the first annotated exon.**

**
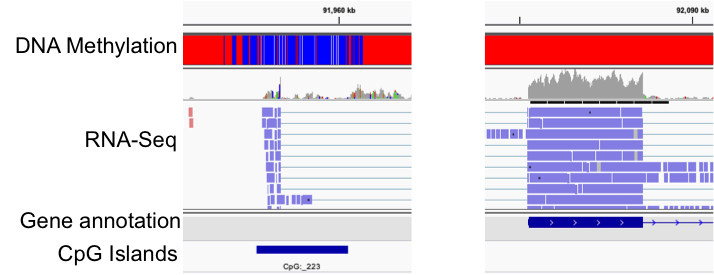
**

**Supplementary Figure S2. A novel promoter of *FAT3* gene.** Spliced reads map to a region 125kb upstream of the annotated *FAT3* first exon. This novel promoter (left) is a hypomethylatedCpG island, a feature characteristic of active promoters whereas the first annotated exon (right), lacking those features, is putatively an internal exon.


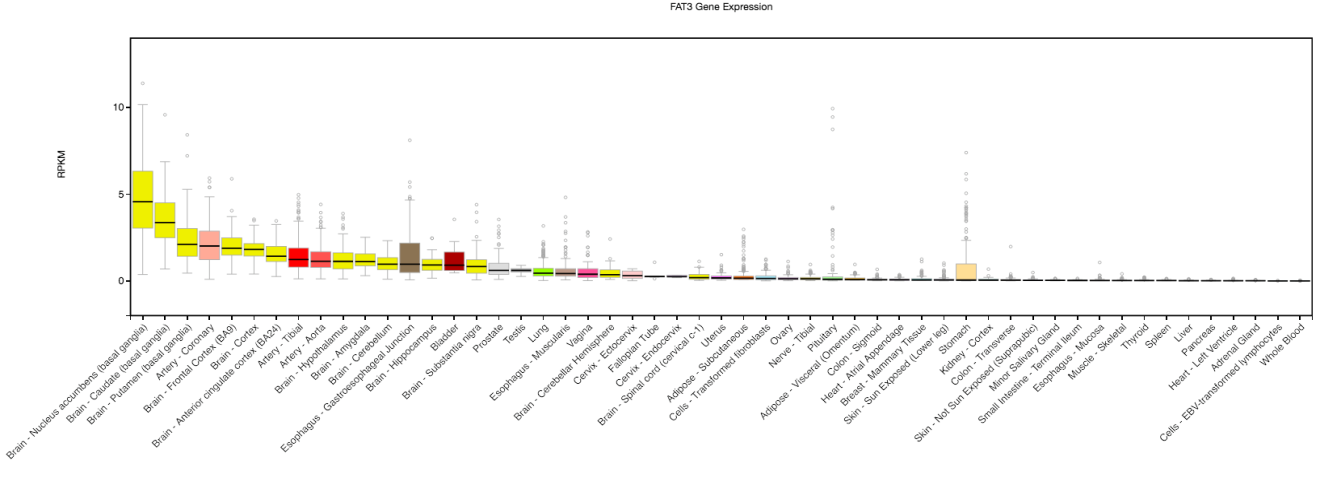


**Supplementary Figure S3. Tissue-specific expression of *FAT3* in GTEx Analysis Release V6 (dbGaP Accession phs000424.v6.p1).** Boxplots show enrichment of *FAT3* expression in brain and artery.

**Supplementary Figure S4. *FAT3* expression levels in primary osteoblasts extracted from 7 scoliotic patients bearing rare variants (from both discovery and replication cohorts) versus 7 controls (trauma patients with no scoliosis).**
